# A Combinatorial Vaccine Containing Inactivated Bacterin and Subunits Provides Protection Against *Actinobacillus pleuropneumoniae* Infection in Mice and Pigs

**DOI:** 10.3389/fvets.2022.902497

**Published:** 2022-06-07

**Authors:** Lijun Zhang, Wentao Luo, Ruyue Xiong, Haotian Li, Zhiming Yao, Wenxiao Zhuo, Geng Zou, Qi Huang, Rui Zhou

**Affiliations:** ^1^State Key Laboratory of Agricultural Microbiology, College of Veterinary Medicine, Huazhong Agricultural University, Wuhan, China; ^2^The Cooperative Innovation Center of Sustainable Pig Production, Wuhan, China; ^3^International Research Center for Animal Diseases, Ministry of Science and Technology (China), Wuhan, China; ^4^The HZAU-HVSEN Institute, Huazhong Agricultural University, Wuhan, China

**Keywords:** *Actinobacillus pleuropneumoniae* (APP), vaccine, bacterins, subunit, immune protection

## Abstract

*Actinobacillus pleuropneumoniae* (APP) is the etiological agent of porcine contagious pleuropneumonia (PCP) that causes great economic losses in the swine industry. Currently, vaccination is still a commonly used strategy for the prevention of the disease. Commercially available vaccines of this disease, including inactivated bacterins and subunit vaccines, have clinical limitations such as side effects and low cross-protection. In this study, a combinatorial vaccine (Bac-sub) was developed, which contained inactivated bacterial cells of a serovar 1 strain and three recombinant protoxins (rApxIA, rApxIIA, and rApxIIIA). Its side effects, immune protection, and cross-protection were evaluated and compared with a commercial subunit vaccine and a commercial trivalent bacterin in a mouse infection model. The results revealed that the Bac-sub vaccine showed no obvious side effects, and induced higher levels of Apx toxin-specific IgG, IgG1, and IgG2a than the commercial vaccines after booster. After a challenge with virulent strains of serovars 1, 5, and 7, the Bac-sub vaccine provided greater protection (91.76%, 100%, and 100%, respectively) than commercial vaccines. Much lower lung bacterial loads (LBLs) and milder lung lesions were observed in the Bac-sub-vaccinated mice than in those vaccinated with the other two vaccines. The protective efficacy of the Bac-sub vaccine was further evaluated in pigs, which showed that vaccinated pigs displayed significantly milder clinical symptoms and lung lesions than the unvaccinated pigs after the challenge. Taken together, Bac-sub is a safe and effective vaccine that could provide high protection against *A. pleuropneumoniae* infection in both mice and pigs.

## Introduction

Porcine contagious pleuropneumonia (PCP), caused by *Actinobacillus pleuropneumoniae* (APP) infection, is one of the most critical respiratory infectious diseases and causes considerable economic losses in the swine industry worldwide (1). PCP is characterized by hemorrhagic, fibrinous, and necrotic lung lesions, and the clinical feature of infected pigs ranges from acute death to chronic pleuritis lung lesions (2). *A. pleuropneumoniae* is classified into at least 19 serovars based on surface polysaccharide antigens (1, 3) and capsule loci (4–6). A series of virulence factors have been described, in which Apx toxins (ApxI-IV) are the most critical ones with different hemolytic and cytotoxic activities (7). Each toxin as a neutralizing antigen is encoded by the *apxCABD* operon and activated by the activator C, and secreted by B and D (8), and expressed by different serovar strains (9–11). Therefore, the nontoxic protoxins ApxIA, ApxIIA, and ApxIIIA are the most crucial protective antigens for a vaccine against this disease.

In the last two decades, most isolates from swine farms in many countries show high resistance to currently recommended antimicrobials for this disease (12–15). Therefore, vaccination has gained attention to prevent this disease. Several vaccination strategies have been confirmed to prevent this disease with varying efficacy. These include three major forms of vaccines: inactivated bacterin, subunit vaccine, and live vaccine. An inactivated bacterin comprising killed bacterial cells from no more than three serovars can generate limited cross-protection and side effects, including fever and long-term tissue swelling at the vaccination site (16, 17). Compared with bacterins, subunit vaccines are mainly based on the immunogenicity of Apx toxins and outer membrane proteins (OMPs) (18), which can reduce clinical signs and lung lesion, but cannot provide complete protection to animals (19). The attenuated live vaccines are developed from a natural non-virulent strain or a genetically modified strain, which can generate heterologous protection, but are not yet approved for use due to safety concerns (20–22).

In this study, to overcome the limitations of existing vaccines, we developed a novel formula of *A. pleuropneumoniae* vaccine consisting of inactivated bacterial cells of a serovar 1 strain and the three recombinant protoxins ApxIA, ApxIIA, and ApxIIIA. We call this combinatorial vaccine “Bac-sub.” Its protective efficacy against the different serovars of *A. pleuropneumoniae* was investigated in a mouse model, which displayed higher cross-protection than either the commercial bacterin (B) or a subunit vaccine (S). The protective efficacy of the Bac-sub vaccine was also confirmed in pigs.

## Materials and Methods

### Bacterial Strains and Growth Conditions

Bacterial strains used in this study are listed in Table 1. APP strains used in this study were isolated from pig farms in China and cultured on tryptic soy agar (TSA) or tryptic soy broth (TSB) (Difco, USA) supplemented with 10 μg/ml of nicotinamide adenine dinucleotide (NAD) and 5% bovine serum at 37°C. *Escherichia coli* DH5α used for gene cloning and *E. coli* M15 used for recombinant protein expression were grown in Luria-Bertani (LB) medium supplemented with 50 μg/ml ampicillin when required.

**Table 1:** Characteristics of bacterial strains and plasmids used in this study.

**Strains or plasmids**	**Relevant characteristics**	**Source/reference**
**Strains**		
4074	*A. pleuropneumoniae* reference strain of serovar 1	From Dr. Pat Blackall
JL03	Local isolate of *A. pleuropneumoniae* serovar 3	Our laboratory
HB01	Local isolate of *A. pleuropneumoniae* serovar 1	This work
HB05	Local isolate of *A. pleuropneumoniae* serovar 5	This work
HB07	Local isolate of *A. pleuropneumoniae* serovar 7	This work
HB10	Local isolate of *A. pleuropneumoniae* serovar 10	This work
DH5α	*E. coli* strain for gene cloning	Trans
M15 (pREP4)	*E. coli* strain for expression with pQE-80L	Qiagen
**Plasmids**		
pQE-80L	Expression vector, ampicillin-resistant, 6×His-tag, 2×lacO	Qiagen
pQE-*apxIA*	Recombinant plasmid for expressing ApxIA	This work
pQE-*apxIIA*	Recombinant plasmid for expressing ApxIIA	This work
pQE-*apxIIIA*	Recombinant plasmid for expressing ApxIIIA	This work

### Preparation of the Recombinant Proteins ApxIA, ApxIIA, and ApxIIIA

The coding sequences of *apxIA* (3,069 bp) and *apxIIA* (2,871 bp) were amplified from the genome of APP 7 strain (GenBank accession no. CP703.1) using the primers apxIAF (5′-ATGGCTAACTCTCAGCTCGA-3′) and apxIAR (5′-TTAAGCTGCTTGTGCTAAAGAA-3′), and apxIIAF (5′-ATGTCAAAAATCACTTTGTCATC-3′) and apxIIAR (5′- TTAAGCGGCTCTAGCTAATTG-3′), respectively. The coding sequence of *apxIIIA* (3,159 bp) was amplified from the genome of APP JL03 strain (GenBank accession no. 778.1) using the primers apxIIIAF (5′- ATGAGTACTTGGTCAAGCATGT-3′) and apxIIIAR (5′-TTAAGCTGCTCTAGCTAGGTTAC-3′). PCR products were inserted into the expression vector pQE-80L *via BamH*I and *Sal*I restriction enzyme sites. The three recombinant proteins were purified from inclusion bodies as follows. *E. coli* M15 carrying each recombinant plasmid was grown to the mid-log phase followed by induction with 0.5 mM of isopropyl β-D-1-thiogalactopyranoside (IPTG) for 6 h at 37°C. Inclusion bodies were dissolved in 8 M urea, purified using nickel beads, and renatured in the buffer containing 100 mM Tris pH8.0, 400 mM L-arginine, 2 mM EDTA, 5 mM reduced glutathione, and 0.5 mM oxidized glutathione. Purified proteins were analyzed by western blotting.

### Hemolytic Assay

To test the hemolytic activity of the recombinant protoxins (rApxIA, rApxIIA, and rApxIIA), we performed the hemolytic assay as previously reported (23). Briefly, the purified protoxins and native toxin ApxIA (1.25 mg/ml) were subjected to a 2-fold series dilution, which were mixed 1:1 with 2% blood solution in a 96-well plate. Native ApxIA purified from the culture supernatant of APP HB10 strain as previously described (23) was used as a positive control. The negative control (NC) was done with lysis buffer only without toxins, while 100% hemolysis was verified with 1% Triton X-100. The plate was incubated at 37°C for 2 h and then centrifuged at 400 × g for 5 min. The optical density of the supernatant at 540 nm (OD_540_) was measured to assess the presence of released hemoglobin.

### Bac-Sub Vaccine Preparation

Cells of APP HB01 strain were subcultured from an overnight culture in TSB medium and grown to the mid-log phase. Bacterial cells were harvested by centrifugation at 7,000 g and 4°C, resuspended in 50 ml of precooled phosphate-buffered saline (PBS) containing 0.3% formaldehyde, and incubated at 37°C for 48 h with shaking. Inactivated bacteria were harvested by centrifugation. Each milliliter of Bac-sub vaccine contains 5.0 × 10^8^ colony forming unit (CFU) of inactivated cells of APP HB01 strain and 125 μg of each of the three recombinant Apx toxins emulsified with the adjuvant Montanide IMS 7 (SEPPIC, France).

### Vaccination and Challenge of Mice

Four- to six-week-old female KM mice were divided into four groups (36 mice in each group) and housed under specific pathogen-free (SPF) conditions. Groups I–III were subcutaneously vaccinated with 0.2 ml of Bac-sub vaccine, a commercial subunit (S) vaccine, and a commercial trivalent bacterin (B), respectively. Group IV was injected with the same volume of PBS mixed with the adjuvant IMS 7, serving as a NC. The S vaccine contains ApxI, ApxII, and ApxIII toxoid, and OMP. The B vaccine contains 5 × 10^8^ CFU/ml of inactivated bacterial cells of serovars 1, 2, and 7 of APP as well as the culture media. All mice were boosted with a second dose 14 days post priming (dpp). The behavior and clinical signs of mice were observed, and body weight was recorded after the priming and boosting. Serum samples were collected by tail bleeding on 0, 7, 14, 21, and 28 dpp and stored at −20°C until use. At 28 dpp, mice in each group were divided into three subgroups (12 mice each) and challenged intraperitoneally with 5 × LD_50_ of HB01 (5 × 10^8^ CFU), HB05 (1.9 × 10^9^ CFU), and HB07 (9.0 × 10^8^ CFU), respectively. The behavior and clinical signs of mice were observed, and the individual survival time was recorded. At 72 h post-challenge (hpc), all survived mice were humanely executed. Lung bacterial loads (LBLs) were determined by bacterial counting on TSA plates supplemented with 10 μg/ml NAD and 5% bovine serum for three of the survival mice. Lung tissues were subjected to histopathological investigation. Lung tissues were fixed in 10% formalin (v/v) overnight, embedded in paraffin, sectioned and mounted on slides, and evaluated with hematoxylin–eosin (H&E) staining by a pathologist who was blinded to all-group delineation (24). Hemorrhage and inflammatory cell infiltration were scored on the following scale, scores from 0 to 3 for each parameter: absent, mild (10% involved), moderate (involving10–50%), and severe (involving 50%).

### Vaccination and Challenge of Pigs

A total of 11 6-week-old APP-free Duroc-Landrace-Yorkshire (DLY) pigs were randomly divided into vaccination group (*n* = 6) and non-vaccination group (*n* = 5). Each pig in the vaccination group was vaccinated intramuscularly with 2 ml of the Bac-sub vaccine and boosted 14 days later. Pigs in the non-vaccination group were injected with 2 ml of PBS mixed with the adjuvant IMS 7. Two weeks after the second vaccination, pigs were challenged intranasally with APP HB01 (7 × 10^8^ CFU per pig). After challenge, the clinical appearance was examined and body temperature was measured every 6 h. The survived pigs were euthanized 72 h after challenge. Lungs were collected and lesions were recorded to give a score of up to five for each apical and intermediate and cardiac lobe, and up to 10 for each of the two larger diaphragmatic lobes (45 in total) (25). Swabs were taken from the upper lobe of each lung and from the cut surface of a bronchial lymph node and swabbed onto TSA plates supplemented with and without 10 μg/ml NAD and 5% bovine serum, respectively, to confirm the presence of APP. Left caudal lung lobes were taken for bacteriological examination as previously described. Sera were collected weekly from the front cavity vein for serological testing.

### Serological Testing

The titer of antibody against the recombinant rApxIA, rApxIIA, rApxIIIA, or whole cells in the mice and pig sera was determined by using enzyme-linked immunosorbent assay (ELISA) as previously described (26). Briefly, 96-well plates were coated overnight with recombinant rApxIA (1.8 μg/well for mice sera and 22.57 μg/well for pig sera), rApxIIA (3.4 μg/well for mice sera and 39.88 μg/well for pig sera), rApxIIIA (2.0 μg/well for pig sera and 11.10 μg/well for pig sera), or the somatic antigen of HB01 (1 × 10^6^ CFU/well for mice sera and 1 × 10^7^ CFU/well for pig sera) at 4°C in which their concentrations were optimized by square titration. The plate was blocked with 5% skim milk (blocking buffer) for 1 h at 37°C, washed five times with 0.05% Tween-20 in PBS (PBST buffer), and stored at 4°C until use. Serially diluted serum samples were incubated with the plate for 1 h at 37°C. After five time washes with PBST buffer, a goat anti-mouse IgG-HRP or rabbit anti-pig IgG-HRP secondary antibody (diluted 1:4,000) (Bioss, Beijing, China) was incubated. To detect IgG subtypes, mouse serum (1:320 dilution) was incubated overnight at 4°C, and goat anti-mouse IgG1-HRP and goat anti-mouse IgG2a-HRP (1:10,000; Bethyl, USA) antibodies were incubated, respectively. Finally, TMB peroxidase substrate (Beyotime, Shanghai, China) was added and incubated in the dark for 30 min. Absorbance was measured at 630 nm using a Tecan M200 plate reader. Serum from naive mice was used as a NC. IgG endpoint titers of mice sera were defined as the reciprocal of the serum dilution when the OD_630nm_ value of the serum was greater than 2.1 times of the preimmunized serum (26). IgG endpoint titers of pig serum were denoted as the first serum dilution having an OD_630nm_ value below the cutoff value, which was determined in our group. The cutoff was calculated as the OD_630nm_ value of a negative reference serum plus two times the standard deviation (SD).

To weigh the strength of the cellular and humoral immunity, we calculated the ratio of ΔIgG1 to ΔIgG2a (the OD_630nm_ value of IgG1or IgG2a in the vaccinated group minus that of the NC group), which was defined as the *R*-value related to the balance of the Th2/Th1 cell response. The *R*-value calculation is not performed when the IgG1 or IgG2a value of the vaccinated group was not significantly different from that of the NC group. *R* > 1.0 indicates a Th2-predominating (humoral) response, and *R* < 1.0 suggests a Th1-predominating (cellular) response. Hemolysin neutralization (HN) titers of pig sera were determined by HN assays and performed exactly as described before (27).

### Statistical Analysis

The mortality, morbidity, and percentage of animals with dyspnea, fever, depression, and anorexia were compared between groups using the Fisher's exact test. Other data are expressed as mean ± standard errors of the mean (SEM) and shown in the graph as mean + SEM and analyzed by the analysis of variance (ANOVA) test. The difference is considered to be statistically significant if the value of *p* ≤ 0.05.

## Results

### Characterization of Recombinant Apx Protoxins

**Figure 1:**
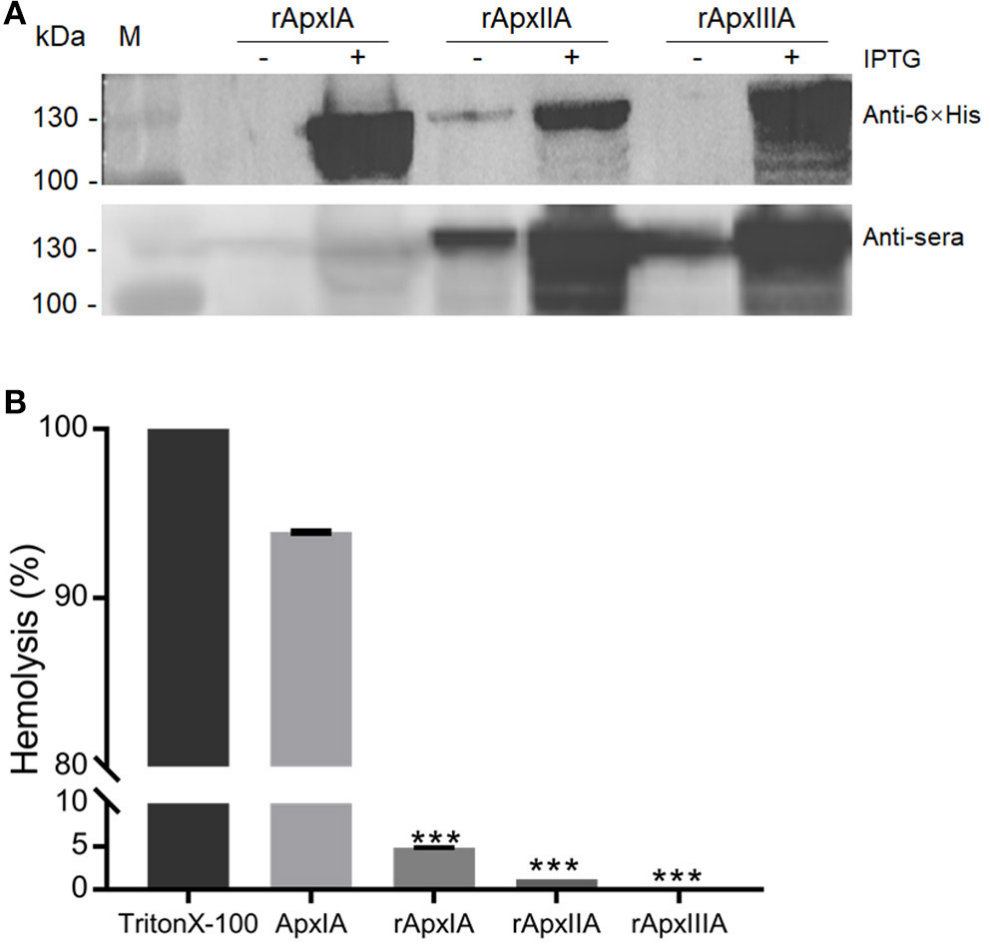
Western blot analysis of the recombinant proteins ApxIA, ApxIIA, and ApxIIIA **(A)** using the 6 × His-Tag antibody (upper panel) and anti-sera of trivalent bacterin-vaccinated pigs (lower panel). Sheep RBCs were lysed with the three protoxins and native ApxIA diluted 2-fold from 125 μg in the hemolytic assay, and 100% RBCs were lysed with Triton X-100 **(B)**. ***p < 0.005 (one-way ANOVA).

The recombinant APP protoxins rApxIA, rApxIIA, and rApxIIIA were successfully purified. These protoxins were probed with the sera recovered from trivalent bacterin-vaccinated pigs, which showed that the recombinant protoxins rApxIIA and rApxIIIA have a good reactivity, while the reaction band of rApxIA is relatively weak due to the relatively lower level of ApxIA antibodies in the sera (Figure 1A). Hemolytic activity was determined with the three recombinant protoxins, which showed that rApxIA, rApxIIA, and rApxIIA had almost no hemolytic activity (Figure 1B).

### Clinical Signs of Mice After Vaccination

After vaccination, no abnormal behavior was observed for mice in groups I (Bac-sub) and II (S), but some mice in group III (B) appeared with rough hair coats and swelling at the injection site within 7 dpp, which were recovered afterward. Moreover, body weight gain (BWG) was similar among groups I, II, and NC during the 28 dpp (*p* > 0.05), whereas the BWG of group III mice was significantly lower than that of the other three groups of mice (*p* < 0.05) (Supplementary Figure S1). These data suggest that the commercial bacterin induced obvious side effects, while our Bac-sub vaccine and the commercial subunit vaccine did not.

### Antibody Response of Mice Upon Vaccination

**Figure 2:**
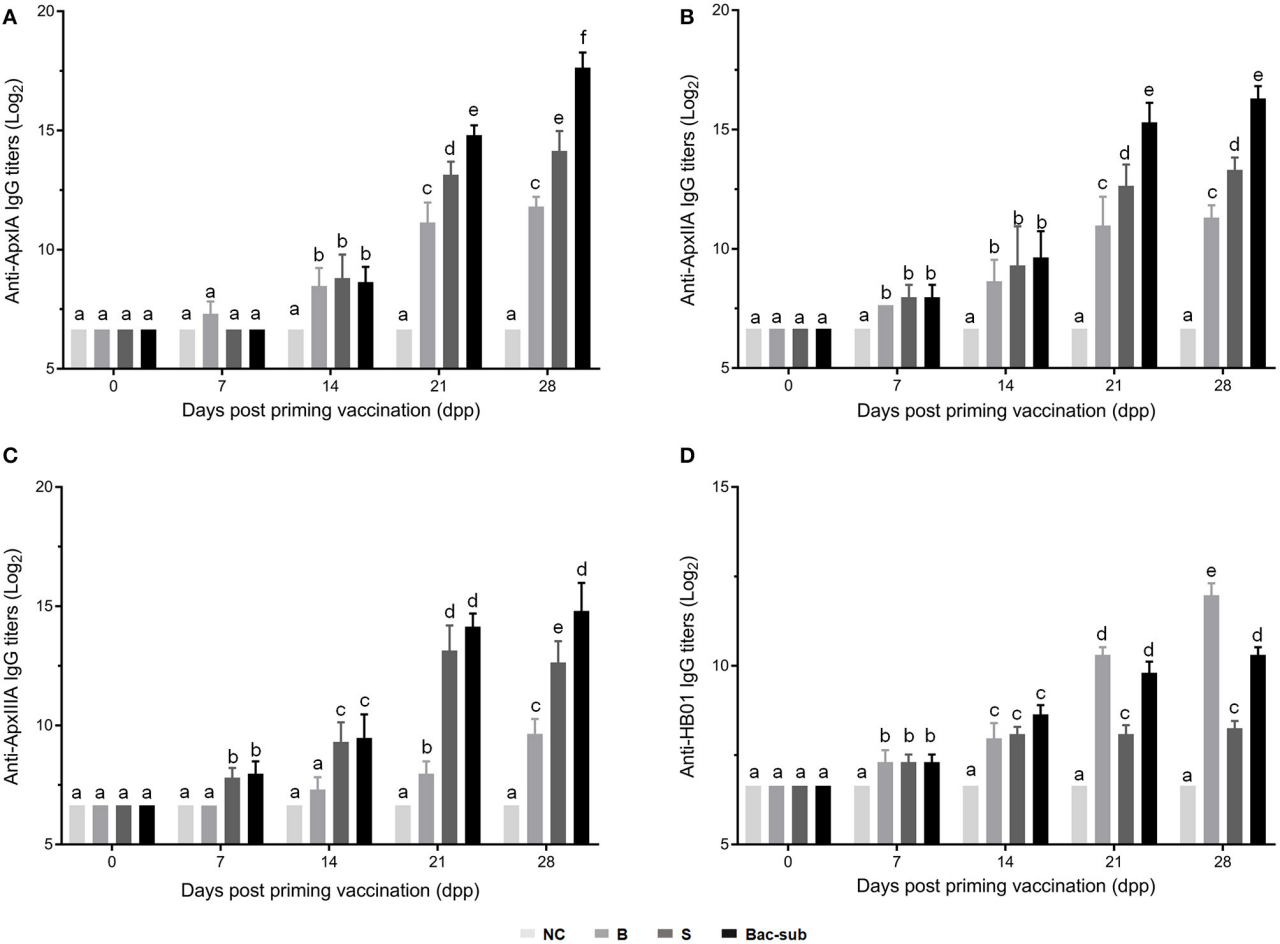
IgG endpoint titers against ApxIA **(A)**, ApxIIA **(B)**, ApxIIIA **(C)**, and HB01 somatic antigens **(D)** in sera of mice vaccinated with the Bac-sub vaccine, subunit (S), bacterin (B), and phosphate-buffered saline (PBS) [negative control (NC)], respectively. When the letters at the top of columns are different, it means that the specific antibody titers are significantly different (*p* < 0.05), otherwise the difference is not significant (*p* > 0.05).

**Figure 3:**
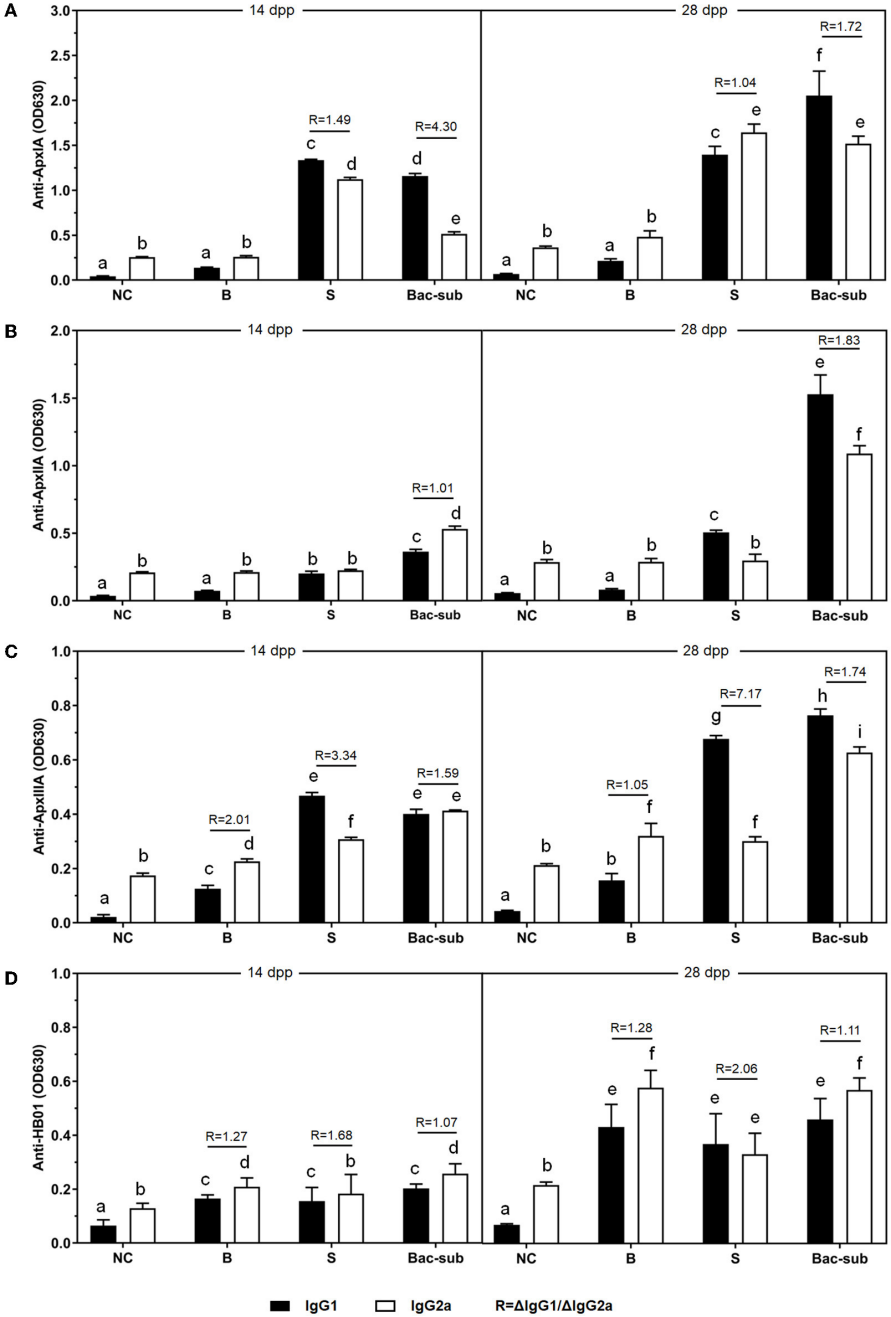
The levels of IgG1 and IgG2a antibodies (OD_630nm_) against ApxIA **(A)**, ApxIIA **(B)**, ApxIIIA **(C)**, and HB01 somatic antigens **(D)** induced by the Bac-sub vaccine, subunit vaccine (S), bacterin (B), and PBS (NC) in mice 7 days after priming and boosting vaccination. *R* = ΔIgG1/ΔIgG2a, (Δ means the value of the vaccinated group minus that of the NC group). Different letters at the top of columns mean that IgG1 or IgG2a titers are significantly different (*p* < 0.05), otherwise not significant (*p* > 0.05).

To investigate the antibody response after vaccination, we determined IgG titers against the three recombinant pro-Apx toxins and the somatic cells of APP HB01 strain in mouse serum samples by ELISA. As shown in Figure 2, at 14 dpp, IgGs specific to rApxIA, rApxIIA, and the bacterial somatic antigen were significantly higher in all the three vaccinated groups than in the control group, indicating successful vaccination. At 2 weeks following boosting (28 dpp), it was shown that the Bac-sub-vaccinated group presented the highest levels of IgGs specific to all three protoxins among the three vaccination groups, and also showed a high level of IgG specific for bacterial antigen (Figure 2). To further characterize the types of immune response induced by the vaccines, the antigen-specific isotypes IgG1 and IgG2a were quantified in the mouse sera at 14 and 28 dpp, respectively. Overall, the dynamics of IgG1 and IgG2a antibodies after vaccination were consistent with those of total IgG antibodies (Figure 3). The levels of antigen-specific IgG1 and IgG2a increased obviously after boosting, and all *R*-values were greater than 1.0 (Figure 3), indicating that both Th1- and Th2-type immune responses were induced by all three kinds of vaccines, and that the Th2 immune response was predominant under these conditions.

### Cross-Protection Against the Challenges in Mice

**Figure 4:**
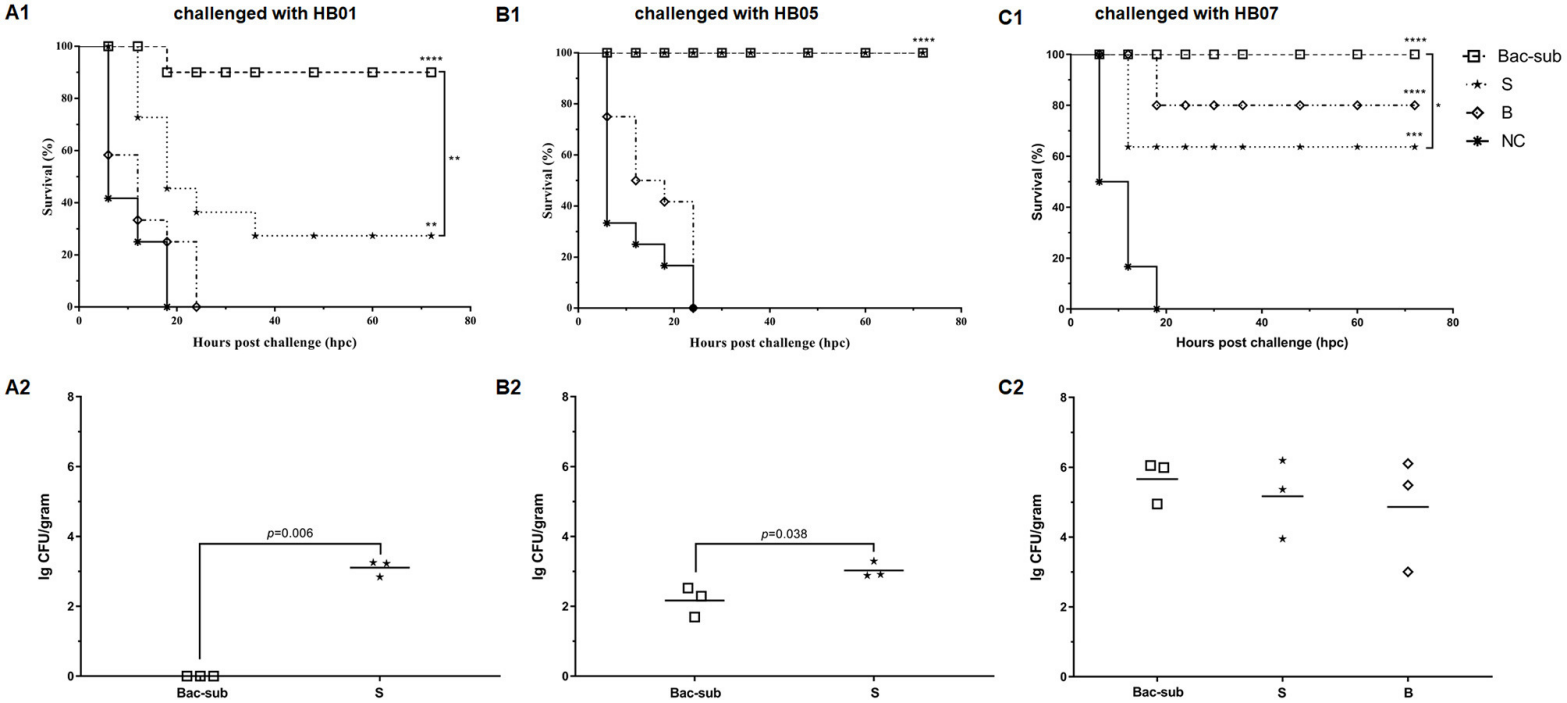
The survival curves of mice **(A1–C1)** and bacterial loads in the lungs of the surviving mice **(A2–C2)**. Mice were vaccinated two times separately with the Bac-sub vaccine, subunit vaccine (S), bacterin (B), and PBS (NC), and then challenged with *Actinobacillus pleuropneumoniae* strain HB01 **(A1,A2)**, HB05 **(B1,B2)**, and HB07 **(C1,C2)**, respectively. The survival of mice and their lung bacterial loads (LBLs) are recorded according to Section “*Materials and Methods*”. Survival rates and LBLs were compared using a one-way ANOVA. *p < 0.05, **p < 0.01, ***p < 0.005, ****p < 0.001.

**Figure 5:**
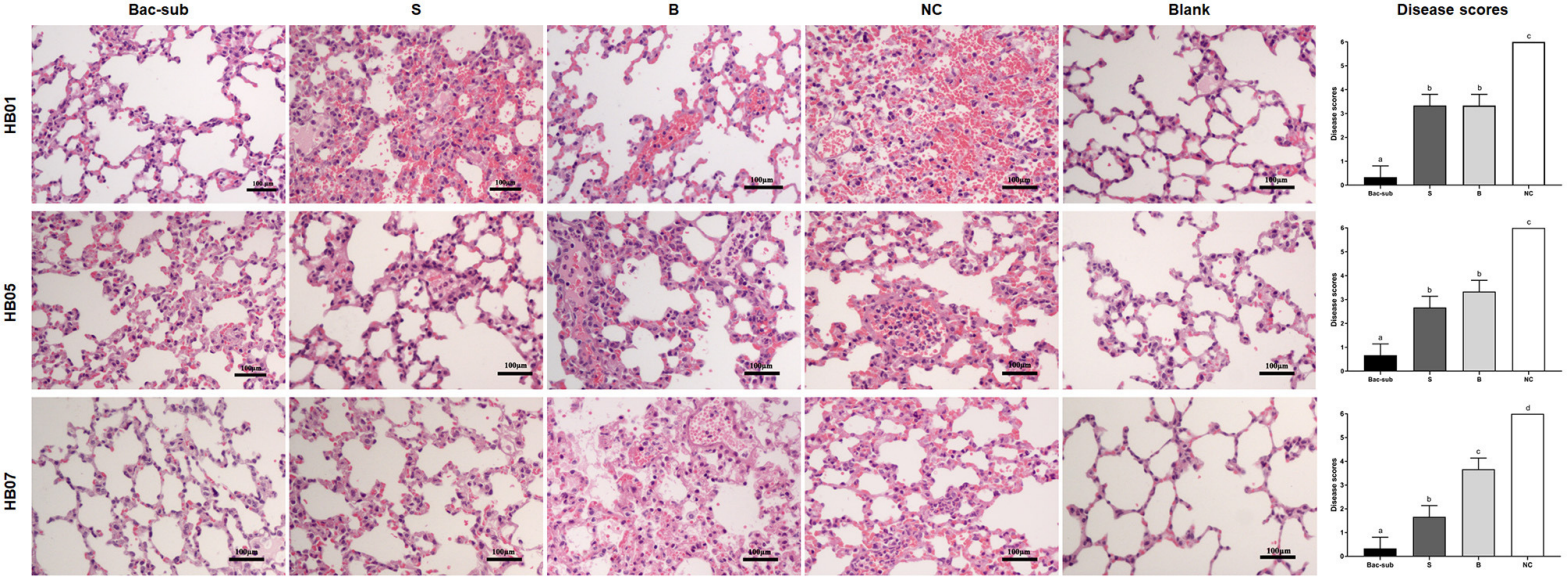
Representative micrographs of lung tissues with hematoxylin and eosin staining and disease scores of lung injury. Mice were vaccinated two times with the Bac-sub vaccine, subunit vaccine (S), bacterin (B), and PBS (NC), and then challenged with *A. pleuropneumoniae* strain HB01, HB05, and HB07, respectively. Blank is the lung of normal mice. Original magnifications × 100. Different letters at the top of columns means significantly different disease scores (*p* < 0.05), otherwise not significant (*p* > 0.05).

To evaluate the cross-protection of the vaccines, the four groups of mice were challenged at 28 dpp with APP of serovars 1, 5, and 7, respectively. It was seen that, after challenge, all mice in the unvaccinated group (NC) died within 24 hpc, whereas most of the vaccinated mice survived within 72 hpc. In detail, the Bac-sub-vaccinated group showed the highest survival, providing 91.66% (11/12) protection against serovar 1 (HB01), and 100% (12/12) against serovar 5 (HB05) as well as serovar 7 (HB07), respectively (Figures 4A1–C1). In comparison, the subunit vaccine (S) only provided 30% (4/12) protection against HB01, 100% (12/12) against HB05, and 66.67% (8/12) against HB07 (Figures 4A1–C1). Bacterin (B) showed the lowest cross-protection, where no protection was achieved after challenge with HB01 and HB05, and 83.33% (10/12) survival was seen against HB07 (Figures 4A1–C1). LBLs of the mice at 72 hpc were isolated and counted (Figures 4A2–C2). APP HB01 could not be isolated anymore in the lungs of Bac-sub-vaccinated mice, whereas 1,400 CFU of HB01 cells were recovered in each gram of lung tissue of the S-vaccinated mice. LBLs of HB05 in B-vaccinated mice were significantly lower than those in S-vaccinated mice (*p* < 0.05). No significant differences were observed in the LBLs of HB07 among vaccinated mice (Figure 4C2). Pathological examinations were further performed to evaluate the degree of lesions in the lungs. Figure 5 shows that the non-vaccination group (UC) showed severe lung lesions, including hemorrhage, inflammatory cell infiltration, and the disappearance of alveoli with a mean disease score of 6.00. Bac-sub-vaccinated mice showed the mildest lung lesions among the three vaccinated groups after challenge with all three serovars (Figure 5). These results suggest that the Bac-sub vaccine provided better cross-protection than the other two commercial vaccines.

### Protection Against the Challenge in Pigs

**Figure 6:**
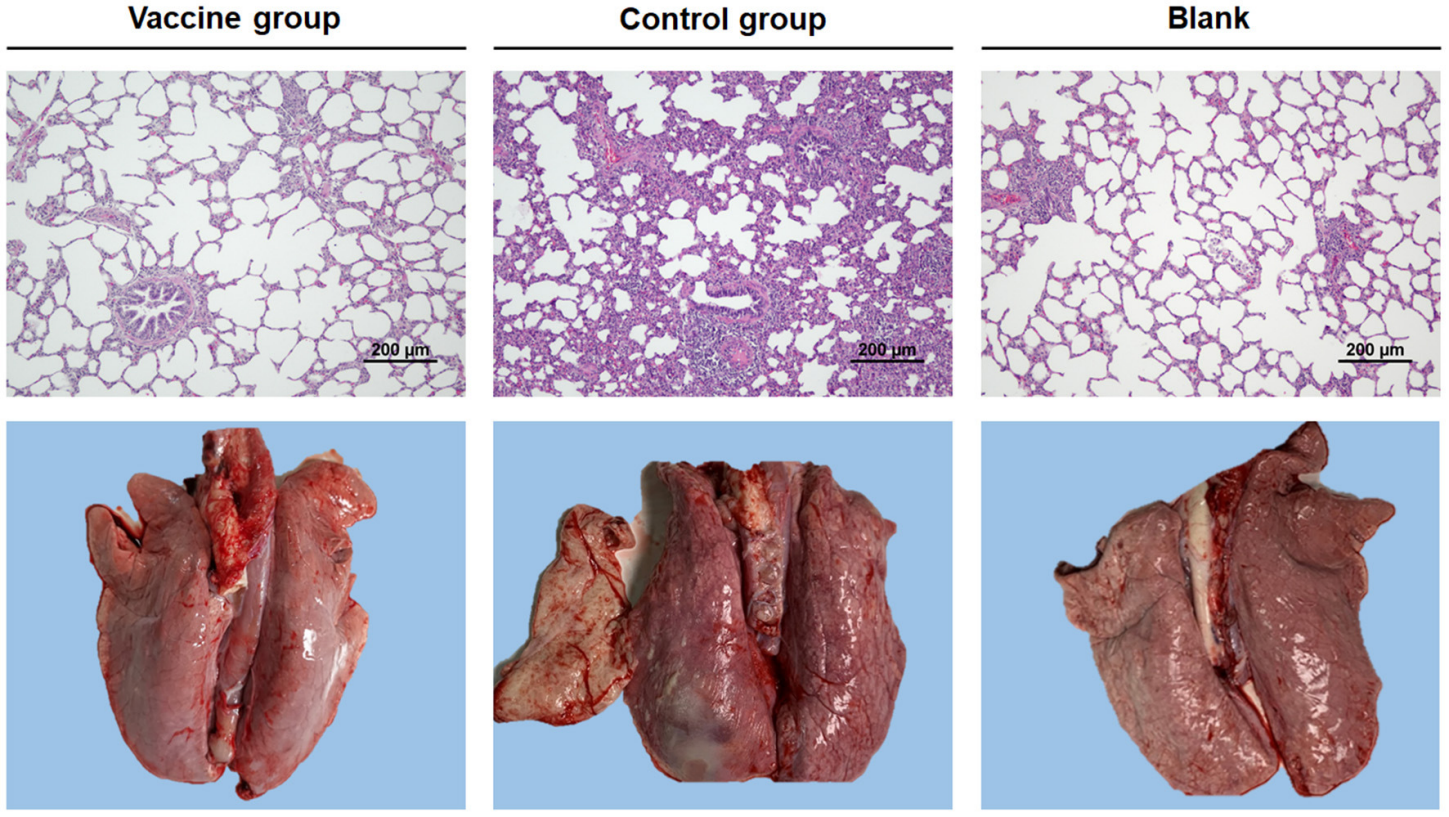
Representative photographs of gross and microscopic lung injury. Pigs were vaccinated two times with bac-subunits (vaccine group) and PBS (control group), respectively, and then challenged with *A. pleuropneumoniae* strain HB01. Blank is the lung of a normal pig. Original magnifications ×200.

We next wondered if the vaccine was also effective in pigs. Thus, the protective efficacy of the Bac-sub vaccine was evaluated using a pig model. After vaccination, no abnormal clinical appearance or swelling at injection sites or other adverse reactions were observed in vaccinated pigs, suggesting no side effects of this vaccine. Following challenge, as seen in Table 2, in the control group one pig died within 24 hpc and the remaining pigs presented fever (>40°C), increased respiratory rate, and coughing within 24 hpc, and two pigs displayed depression and anorexia at 72 hpc. In contrast, in the vaccinated group (*n* = 6), no pigs showed dyspnea and fever, and only one pig presented depression and poor appetite at 24 hpc, which was recovered at 72 hpc. At necropsy, classical hemorrhagic necrotizing pneumonia and fibrinous pleuritis were observed in the lungs of control pigs (mean score of 21.3). In comparison, three pigs in the vaccination group showed only mild hemorrhagic and pneumonia and the remaining three pigs presented almost no obvious lung lesions (mean score of 2.7). Lung histopathology further showed that significantly milder alveolar epithelial hyperplasia and inflammatory cell accumulation and lighter lung lesion in sections were observed in vaccinated pigs than in control pigs (Figure 6). The bacteria burden was also significantly lower in the lungs of vaccinated pigs than in unvaccinated pigs (Table 2). These results suggest that the Bac-sub vaccine provided good protection against APP infection in pigs.

**Table 2:** The results of challenge with *Actinobacillus pleuropneumoniae* serovar 1 in vaccinated and unvaccinated control pigs.

**Treatment**	**Control group** **(*n* = 5)**	**Vaccine group** **(*n* = 6)**
Mortality^a^ (%)	20 (1/5)	0
Morbidity (%)	80 (4/5)	16.6 (1/6)^*^
Animals with dyspnea^b^ (%)	80 (4/5)	0^*^
Animals with fever^b^, ^c^ (%)	60 (3/5)	0
Animals with depression and anorexia^b^ (%)	40 (2/5)	16.6 (1/6)
Median lung lesion score	21.3 ± 6.26	2.7 ± 2.92^*^
Mean bacterial titer in caudal lung lobes (CFU/g)	5.5 × 10^4^ ± 9.84 × 10^4^	23 ± 49.9^*^

a *Morbidity is given as the percentage of animals with increased respiratory rate and/or fever*.

b *only those pigs that survived more than 24 h after challenge were included*.

c *fever is defined as a body temperature of 40°C*.

* *p < 0.05*.

## Discussion

*Actinobacillus pleuropneumoniae* utilizes multiple virulence factors for pathogenesis. Adhesins, including fimbriae and pili, are involved in colonization (28). Several nutrient acquisition and uptake systems are reported to be necessary for growth within the host (29). The capsular polysaccharide on the surface of bacteria contributes to immune evasion (30). Apart from these surface-expressed virulence factors, APP secretes four kinds of exotoxins ApxI–IV. These Apx toxins are crucial virulence factors for APP to develop lung lesions (11). Based on the mechanism of pathogenesis, two major types of vaccines have been developed to prevent APP infection, including bacterins and subunit vaccines. However, both types of vaccines have their limitations. Bacterins can be composed of inactivated bacterial cells of no more than three serovars. This is insufficient to provide cross-protection against such a pathogen comprising more than 19 serotypes (31). Subunit vaccines consisting of conserved antigen proteins among different serotypes can reduce clinical signs and lung lesion; however, it is unable to provide complete protection (19). A vaccine containing both inactivated bacteria and Apx toxins is expected to have better efficacy (32). Therefore, we developed a combinatorial vaccine containing inactivated bacterial cells of a serovar 1 strain and three recombinant protoxins (rApxIA, rApxIIA, and rApxIIIA).

Using a mouse model, we compared the protective efficacy of our combinatorial vaccine (Bac-sub) with the commercial bacterin (B) and a commercial subunit vaccine (S). Compared with the group injected with the B vaccine, which caused obvious side effects, such as abnormal behavior, swelling at the injection site, and lower body weight gain (Supplementary Figure S1), our Bac-sub vaccine did not induce any adverse reaction in mice. Side effects of bacterins have also been observed in previous studies (18). This may be due to a larger amount of somatic cells in the B vaccine, which comprises APP cells of three serovars. Our Bac-sub vaccine contains only one-third of the amount of bacterial cells. Also, the B vaccine showed less cross-protection than the Bac-sub vaccine. Regarding the S vaccine, it provided some degree of cross-protection which, however, was less than that of the Bac-sub vaccine (Figure 4). It can be seen from Figures 2, 3 that a lower level of IgG titers was generated in the mouse in group II (S) than in group I (Bac-sub). This was reasonable because only one somatic antigen OMP was present in this vaccine. This suggests that the combination of recombinant Apx toxins and somatic cells is a good strategy to develop APP vaccines.

When choosing the pro-Apx toxins in the combinatorial vaccine formula, recombinant ApxIA, ApxIIA, and ApxIIIA, but not ApxIVA, were included. This is because ApxIVA is present in all serovars of APP and is expressed only during the process of infection (33). Therefore, it is an important diagnostic marker to differentiate infected from vaccinated animals. This DIVA concept is essential for disease eradication programs (34).

In terms of immune responses, all three kinds of vaccines tested in this study could stimulate both Th1 and Th2 immune responses, but Th2 responses were much stronger than Th1 responses (Figure 3). As we know, induction of Th1 (cellular) immune response is significant for a vaccine. It is still a challenge to design a vaccine that can stimulate a high level of Th1 response against an extracellular bacterial pathogen. The identification of Th1-type antigens and the development of novel adjuvants or antigen carriers might be the directions worthy of further study.

Based on the induced protection of the Bac-sub vaccine in mice, the immune efficacy of the vaccine was further evaluated in pigs. To follow the way of natural infection, we carry out quantitative intranasal inoculation with the aid of a nasal spray. Compared with the non-vaccination control group, pigs in the Bac-sub group showed much milder lung lesions, and most of the APP pathogens in the bronchi, lymph nodes, and lungs were cleared. As shown in Supplementary Table S1, the results showed high levels of neutralizing antibodies of hemolysin ApxIA and antibody levels specific to three Apx and bacterial antigens of HB01 after boosting immunization (Supplementary Table S1), which verified the immune protection efficacy of the Bac-sub vaccine. The results of clinical appearance and histopathological studies revealed that the Bac-sub vaccine could reduce mortality and also greatly reduce morbidity.

In conclusion, this study developed a combinatorial vaccine consisting of bacterin and subunits (Bac-sub) against *A. pleuropneumoniae*. The combinatorial vaccine overcame the limitations of bacterins and subunit vaccines and could induce much higher immune efficacy and protection against the challenge of heterologous APP in mice and challenge of homologous APP in mice and pigs. It is a promising vaccine candidate for PCP.

## Data Availability Statement

The original contributions presented in the study are included in the article/Supplementary Material, further inquiries can be directed to the corresponding author/s.

## Ethics Statement

The animal study was reviewed and approved by all animal experiments were carried out according to the protocols approved by the Scientific Ethics Committee of the University (No. HZAUMO-2018-009 for mice and HZAUSW-2019-023 for pigs).

## Author Contributions

RZ and LZ designed the research idea. LZ, WL, and RX performed the experiments. ZY, WZ, and HL were involved in sample collection and laboratory analyses. LZ, GZ, and QH performed the statistical analyses and drafted this manuscript. LZ, QH, and RZ edited and reviewed this manuscript. All authors made a significant contribution to the study concept and approved the final version for submission.

## Funding

This work was supported by the Wuhan Science and Technology Planning Project (CN) (2018020401011300).

## Conflict of Interest

The authors declare that the research was conducted in the absence of any commercial or financial relationships that could be construed as a potential conflict of interest.

## Publisher's Note

All claims expressed in this article are solely those of the authors and do not necessarily represent those of their affiliated organizations, or those of the publisher, the editors and the reviewers. Any product that may be evaluated in this article, or claim that may be made by its manufacturer, is not guaranteed or endorsed by the publisher.
