# Supplementary material for: A Combinatorial Vaccine Containing Inactivated Bacterin and Subunits Provides Protection Against Actinobacillus pleuropneumoniae Infection in Mice and Pigs
Source: Front Vet Sci. 2022 Jun 7;9:902497. doi: 10.3389/fvets.2022.902497 (PMC9212066; doi:10.3389/fvets.2022.902497)
Supplement: Supplementary file 1 [file Image_1.pdf]

Supplementary Figure

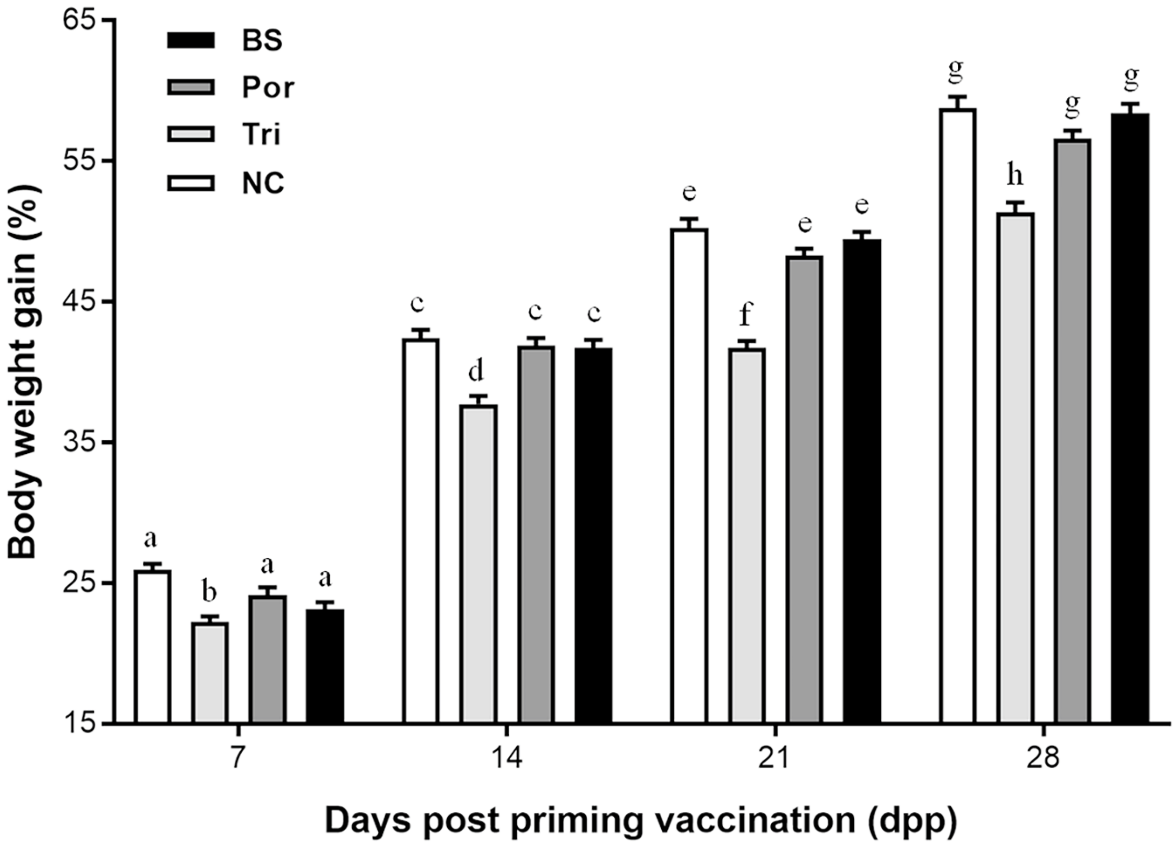

Figure S1. The body weight gain of mice after vaccination. The body weight gain of the mice which were vaccinated with the Bac-sub vaccine, subunit vaccine (S), bacterin (B), and PBS (NC) was calculated as the percentage of body weight compared to the mice weight before vaccinate. When the letters on the top of columns are different means the specific antibody titers are significantly different ($p < 0.05$), otherwise the difference is not significant ($p > 0.05$).
