# Supplementary material for: A Combinatorial Vaccine Containing Inactivated Bacterin and Subunits Provides Protection Against Actinobacillus pleuropneumoniae Infection in Mice and Pigs
Source: Front Vet Sci. 2022 Jun 7;9:902497. doi: 10.3389/fvets.2022.902497 (PMC9212066; doi:10.3389/fvets.2022.902497)
Supplement: Supplementary file 2 [file Table_1.DOCX]

## Supplementary Table

**TABLE S1.** Results of IgG titers against ApxIA, ApxIIA, ApxIIIA, and HB01 somatic antigens and ApxI neutralization titers (HN) in pigs on 21 and 28 dpp

| Treatment | Median IgG Titers | | | | | | | | Median ApxI Neutralization Titers | |
| --- | --- | --- | --- | --- | --- | --- | --- | --- | --- | --- |
|  | Anti-ApxIA | | Anti-ApxIIA | | Anti-ApxIIIA | | Anti-HB01 | |  |  |
|  | 21 dpp | 28 dpp | 21 dpp | 28 dpp | 21 dpp | 28 dpp | 21 dpp | 28 dpp | 21 dpp | 28 dpp |
| Vaccine group (n=6) | 853±301^*^ | 1813±776^*^ | 1066±301^*^ | 1600±715^*^ | 853±301^*^ | 1493±477^*^ | 560±178^*^ | 453±194^*^ | 3380±1825^*^ | 7846±4784^*^ |
| Control group (n=5) | 66±18 | 60±20 | 53±19 | 46±15 | 73±15 | 66±19 | 66±19 | 60±20 | 1619±356 | 2095±609 |

dpp: days post priming vaccination. Data of each group represent the means ± SEM. For 21 dpp and 28 dpp IgG titers and HN titers, * indicates the medians are significantly different (*p* < 0.05) between vaccinated pigs and non-vaccinated pigs.
